# What determined match outcomes at the 2026 FIFA World Cup? An explainable machine learning analysis of penalty area precision and composure under pressure

**DOI:** 10.5114/biolsport.2026.164000

**Published:** 2026-08-11

**Authors:** Ismail Dergaa, Ryland Morgans, Karim Chamari

**Affiliations:** 1 High Institute of Sport and Physical Education of Ksar Said, University of Manouba, Tunisia; 2 School of Sport and Health Sciences, Cardiff Metropolitan University, Cardiff, United Kingdom; 3 Naufar Center, Doha, Qatar

**Keywords:** Conformal prediction, Football, Gradient boosting, Neural networks, Performance indicators, SHAP, Soccer

## Abstract

The 2026 FIFA World Cup was the first edition played by 48 teams across 104 matches. We analysed 142 official FIFA metrics per team per match, including line breaks, pressing events and receptions between the lines, none previously analysed at a World Cup. Win and loss performances were compared with Cliff’s delta under false discovery rate control; 86 of 125 screened metrics differed. Outcomes were modelled with five algorithms under repeated stratified cross-validation, using team-minus-opponent differentials. Explainability used SHapley Additive exPlanations (SHAP), and uncertainty used split-conformal analysis, a first World Cup combination. On-target attempts inside the penalty area showed the largest single effect (winners 4.4 versus losers 1.7; delta = 0.68). Win probability crossed 50% at 2.9 and 75% at 4.3 of these attempts. The regularised differential logistic model reached area under the curve 0.946 (accuracy 0.875), read descriptively because features exceed matches. Walking distance ranked first in averaged SHAP importance in the gradient boosting model; its strongest interactions joined total attempts on target (0.79) and on-target attempts inside the penalty area (0.71), the second-ranked feature. Teams dominating all five leading duels won 92% of decided matches; teams dominating none won 13%. Conformal coverage averaged 0.885 at the 90% target; 15% of matches remained uncertain. Finishing precision was the most consistent discriminator across stratifications (balanced-pairing delta = 0.53 inside the box, -0.54 conceded). Pressure-resistant possession showed strong overall and knockout-stage associations (delta = 0.56 and 0.65). Building these two capacities is the most defensible practical priority, pending prospective confirmation.

## INTRODUCTION

Match analysis has moved from manual notation to large-scale positional and event data, and this shift has changed how team performance is understood [1]. Analytical systems now frame a football match as an interaction of technical, tactical and physical layers rather than a list of isolated counts [2]. Machine learning has entered professional football, and its applications now include match outcome prediction, tactical pattern detection and player evaluation [3]. Through this growth, the central applied question has remained unchanged for two decades. Which performance behaviours separate winning from losing at the highest level of football?

The FIFA World Cup has served as the reference for this debate. Shots on target discriminated successful from unsuccessful teams across the 2002 to 2010 tournaments [4], and modelling of the 2014 FIFA World Cup confirmed shots on target as the dominant single predictor of match outcome [5]. Further research into the 2014 and 2018 tournaments added possession quality, shot efficiency and defensive compactness to the winning profile [6]. Goalscoring pattern analysis at the 2018 tournament emphasised attacking speed, the speed of the attacking move preceding the goal, and set plays (goals arising from corners, free kicks and penalties) [7]. Physical outputs such as total and high-intensity distance showed weaker and less consistent relationships with performance, with style-related effect sizes of 0.33 to 0.47 against up to 1.27 for technical actions [8]. Longitudinal cross-tournament modelling from 1994 to 2022 identified shooting efficiency, the share of attempts converted into goals, as the most stable performance determinant [9]. More recently, machine learning entered team analysis at the 2022 FIFA World Cup [10, 11]. Ensemble models explained with SHapley Additive exPlanations (SHAP) identified attempts on target, shooting opportunities and ball progressions as the most influential features [10]. Discriminant models of the same tournament highlighted forced turnovers (possession regains forced by defensive action), pressing actions (defensive engagements of the opponent in possession) and whole-match running outputs as contributing factors to successful outcomes [11]. Defensive organisation, expressed through the structure and positioning of the defending team during defensive transitions [12], and passing-network structure, describing player prominence and team interaction patterns [13], completed a thorough analytical account of the 32-team format.

The 2026 FIFA World Cup was the first tournament played under a 48-team format. The new structure comprised 104 matches, added a round of 32 and produced wider within-group ranking gaps than any previous competition, a structural concern raised repeatedly in the tournament design literature [14, 15, 16].

This new format arrived together with a new generation of data, and the combination defines the novel ground of our study. FIFA now publishes an enhanced team metric set for every match, and its most informative additions describe behaviour that older data feeds could not see [17]. Line breaks count the passes and carries that eliminate an opposing defensive line. Receptions between the lines count the moments a player accepts the ball in the space behind midfield pressure. Pressure events record how often actions are attempted and completed while an opponent closes down. Importantly, none of these metrics appears in any peer-reviewed World Cup analysis summarised above, so this study provides their first tournament-level examination. The analytical toolkit has also advanced in parallel. SHAP interaction decomposition [18] can show how two metrics operate together rather than rank them in isolation, an approach recently extended to offensive transitions in elite women’s football [19]. Conformal prediction [20] attaches a calibrated statement of certainty to every match-level prediction. Neither method had been applied to football tournaments. The expanded field itself raises the analytical value of a single edition: 104 matches form the largest single-tournament sample in World Cup history. Together, these openings allow the first test of whether the determinants identified in the 32-team era still hold when the field widens, whether they hold equally in the group stage and the expanded knockout programme, and whether they hold when evenly matched teams meet.

The primary aim of the present study was to identify the performance metrics that separated winning from losing teams across all matches of the first 48-team World Cup, using the complete official FIFA metric set. Three secondary aims followed: (1) to quantify how these determinants combine, through interaction analysis, dominance profiles and practical thresholds; (2) to test whether the determinant structure differed between stages and between balanced and unbalanced pairings; and (3) to characterise the styles of winning performances and the certainty of outcome prediction. Linked to the primary aim and based on the 32-team evidence, we hypothesised that on-target attempts inside the penalty area would remain the strongest single discriminator. We further hypothesised that winners would record higher values than losers on the newly available pressure-related metrics.

## MATERIALS AND METHODS

### Study design and data source

This was an observational analysis of the complete 2026 FIFA World Cup, played in Canada, Mexico and the United States from 11 June to 19 July 2026. The observational analysis is reported in accordance with the STROBE statement [21], and the prediction model is reported following the TRIPOD+AI statement [22]; the completed checklists are provided as supplementary files. All team-level match statistics were extracted from the official FIFA match centre (fifa. com), which publishes the FIFA Football Intelligence metrics for all matches [17]. Independent validation work supports this class of data: optical tracking systems of the type underpinning the physical metrics show football-specific validity for position-derived outputs [23], and professionally collected live football event statistics show high inter-operator reliability [24]. Data were extracted programmatically between 14 and 20 July 2026, and the complete feed was re-extracted after peer review on 5 August 2026. The two extractions matched exactly for all 206 team-match rows of matches 1 to 103; the only differences concerned the final, whose provisional values were replaced by the finalised advanced metric panel that FIFA published after the original window. Access and use were limited to non-commercial academic research, consistent with the licence granted in sections 5.3 and 6.4 of the FIFA terms of service (version of 27 January 2026); dated screenshot and verbatim text archives of these terms, captured on 20 July 2026, are provided as a supplementary evidence file.

### Match sample and outcome coding

The tournament comprised 104 matches: 72 in the group stage, 16 in the round of 32, eight in the round of 16, four quarter-finals, two semi-finals, the third-place play-off match and the final. Each match contributed two team-match observations. Match outcome was coded as win, draw or loss at the end of play, extra time included. Knockout matches decided by penalty shootout were coded as draws for performance analysis, with the penalty shoot-out score recorded separately; four matches ended this way. Across the tournament, 80 matches produced a winner (hereafter, decided matches) and 24 were drawn. One group match (Belgium versus Egypt) lacked the complete advanced-metric panel in the FIFA feed (still unpublished as of 5 August 2026) and was excluded from modelling. That match was drawn, so all 80 decided matches remain available, leaving 103 complete-panel matches (206 team-match observations; 160 win or loss performances).

### Performance metrics and leakage control

FIFA published 142 team metrics per match across attacking, distribution, defending, goalkeeping, discipline, physical and phase-of-play categories. Twelve metrics that encode the match outcome, including goals, goals conceded, assists, clean sheets, scored penalties, goalkeeper save percentage and expected goals, were removed prior to modelling to prevent target leakage, the inclusion of predictors that contain the outcome itself. Four context variables were also removed: matches played, substitutions in, substitutions out and time played. Metrics missing in more than 5% of observations were excluded (one metric); after this exclusion, no missing value remained in the modelling set, so no imputation was applied. In sum, of the 142 published metrics, removing the 12 outcome metrics, the four context variables and the one high-missingness metric (goals conceded from attempts against) left 125 metrics for the univariate comparisons. Collinear metrics were reduced by removing one member of every pair with an absolute Pearson correlation above 0.90, leaving 101 metrics for the paired specification. In the differential specification, the two conceded-attempt metrics become exact negatives of their attacking counterparts and were removed, leaving 99 differential features for the primary specification.

### Data verification and quality audit

Every extracted value passed a three-layer audit prior to analysis. Scores were mirror-checked across the paired rows of all matches. The goals metric was checked against the official score in every row. Team aggregates for seven headline metrics (goals, assists, attempts, attempts on target, expected goals, corners and possession) were cross-checked against FIFA team statistics for all 48 teams, allowing 336 comparisons: 333 matched exactly at the mid-tournament check. Each deviation traced to a match FIFA had not yet finalised and cleared once that match was finalised, leaving only the Belgium-Egypt feed open. All 208 team-match rows were re-verified against the live FIFA feed on 5 August 2026: only the two rows of the final had been revised by FIFA in the interim, and the revised values were adopted; the remaining 206 rows matched the original extraction exactly, an incidental stability check on the source feed. Knockout bracket progression, including shootout advancement, was verified against the official bracket. Five rows carried an attempt subcategory anomaly of one unit in the FIFA source feed and were flagged for sensitivity analysis.

### Statistical analysis

Because the two performances of a match are paired, win and loss team performances were compared metric by metric with the Wilcoxon signed-rank test on within-match differences, and effect size was expressed as Cliff’s delta [25]. False discovery rate was controlled with the Benjamini-Hochberg procedure at q < 0.05, where q denotes the false discovery rate adjusted probability [26]. An unpaired Mann-Whitney U test served as a sensitivity check and gave consistent results. The same analysis was repeated within four strata: group stage, knockout rounds, balanced pairings and unbalanced pairings. Pairing balance used the official FIFA draw pots of December 2025, with a pot difference of 0 or 1 defining balanced and 2 or 3 defining unbalanced matches. Alpha was 0.05 throughout. Analyses used Python 3.11 with scikit-learn 1.8.0, SciPy 1.17.1 and SHAP 0.51.

### Machine learning pipeline: differential modelling and validation

The primary specification modelled each decided match as one observation, with features defined as the difference between the two teams on every metric and the reference side assigned at random to remove listing bias. This differential design removes the statistical dependence between the two rows of the same match. Five algorithms were compared: L2-regularised logistic regression, random forest [27], gradient boosting [28], and a support vector machine with radial kernel. A shallow neural network (multilayer perceptron, hidden layers of 32 and 16 units) completed the set, testing whether nonlinear representation learning adds value at this sample size. A nested cross-validation with an inner tuning grid quantified the effect of hyperparameter search for gradient boosting. Performance was estimated with repeated stratified cross-validation (five folds, five repeats for the primary model, three repeats elsewhere), reporting area under the receiver operating characteristic curve, accuracy and F1. Default configurations were reported for all untuned models, and the tuned gradient boosting used the nested design, avoiding the selection bias described for small-sample model tuning [29]. The paired two-row specification and a three-class win-draw-loss model served as robustness checks. For replication: metric screening (the missing-rate filter and the collinearity filter) used no outcome information and was applied once to the full dataset prior to modelling. Feature scaling was fitted inside each training fold through the modelling pipeline. Tied metric values were left untransformed and no power transformation was applied. The differential outcome was balanced by construction (40 wins in 80 matches after random reference-side assignment), so no class weighting was used in any model. A census of all tournament matches was analysed by design, so no a priori sample size calculation applied. With 40 events among 80 matches and 99 candidate features, the events-per-variable ratio is 0.4, far below conventional guidance for unpenalised models. We therefore used L2 regularisation, which shrinks coefficients and tolerates more features than observations, kept every screening step free of outcome information, and treated model discrimination as descriptive, with the effect-size analysis as the primary evidence.

### Explainability, interactions and uncertainty quantification

Explanation used SHAP values on the gradient boosting differential model, with global importance taken as the mean absolute SHAP value per feature, averaged across 25 random reference-side assignments to remove dependence on the arbitrary assignment [18]. The gradient boosting model was chosen for explanation, despite the higher discrimination of the logistic model, because exact SHAP values and pairwise SHAP interaction values are computable for tree ensembles at feasible cost. The interaction decomposition central to the secondary aims is not available with comparable exactness for the other algorithms. The reported SHAP ranking therefore describes importance within the gradient boosting model, not a model-independent hierarchy of determinants. SHAP values are expressed on the log-odds scale of the gradient boosting model, and the explainer baseline equals the mean model output over the analysis set, so each value reads as a shift in log-odds relative to that baseline. Pairwise interaction structure used SHAP interaction values, which decompose each prediction into main and interaction contributions. Prediction uncertainty used split-conformal analysis at a 90% coverage target, with the regularised differential logistic regression model generating the conformal scores. In each repetition, decided matches were split into training, calibration and held-out test parts; the model was fitted on the training part and nonconformity scores were computed on the calibration part. The nonconformity score of a match was defined as one minus the predicted probability assigned to its true outcome. The empirical quantile of the calibration scores at the nominal level set the threshold. Each test match then received a prediction set containing every outcome whose score fell within it, one or both outcomes in practice [20]. Matches with two-outcome sets were read as statistically uncertain; coverage was evaluated on held-out test splits across 20 repetitions and across five nominal confidence levels (75% to 95%). The stability of the SHAP importance ranking was probed by correlating each assignment’s ranking with the averaged ranking (Spearman correlation), and SHAP dependence plots with interaction colouring visualised the leading feature pair in one representative assignment.

### Winning profiles: factor counts, thresholds and profile clustering

Three complementary profile analyses were performed. First, five key duels were defined from the leading effect sizes, taking one metric per family and excluding conceded mirrors and collinear duplicates: on-target attempts inside the box, possession, distributions completed under pressure, receptions between the lines and ball recovery time. For each decided match, a team won a duel when it exceeded its opponent on that metric, or recorded the faster recovery time; the count runs from zero to five and describes dominance across metrics, not chronological duels within the match. This selection was outcome-informed and made once on the complete dataset, outside the cross-validation loop. The five-metric model reported under Classification performance across algorithms and specifications is therefore a post hoc descriptive analysis, not a pre-specified independent test. Each team-match was scored by the number of duels won, and win percentage was computed per count. Second, univariate logistic curves provided the metric values at which win probability crossed 50% and 75%, providing practical benchmarks. Third, winning performances were clustered with k-means on fifteen standardised style metrics, with the cluster number selected by silhouette [30] over k = 2 to 5. A shallow decision tree (depth 2, minimum leaf 15) provided interpretable combination rules as a complement to the ensemble models.

### Ethics statement

Ethical approval was not applicable. The study analysed publicly available team-level match statistics published by FIFA at fifa.com. No human participants were involved and no individual player data were processed; the analysis constitutes secondary use of publicly available aggregate information.

### AI usage statement

In preparing this manuscript, the first author used ChatGPT version GPT-5.6 Thinking (OpenAI) on 19 July 2026 to improve the fluency and grammatical quality of selected passages of the initial draft. The tool was not used for data analysis, interpretation or generation of scientific content. The first author manually revised every passage before circulating the draft, and the co-authors then reviewed and improved the manuscript through manual revision only, without the use of any generative artificial intelligence tool. All authors take full responsibility for the accuracy, integrity and scientific validity of the work [31, 32].

## RESULTS

### Match sample and descriptive statistics

The tournament produced 308 goals across its 104 matches: 80 decided and 24 drawn, with four knockout draws resolved by shootout. The bronze final alone produced ten goals (France 4-6 England) and the final one (Spain 1-0 Argentina). Twelve of 71 decided pairings between teams from different draw pots ended as wins for the lower-seeded team (17%). The modelling set comprised the 103 complete-panel matches with 80 decided. Table 1 summarises the sample.

**TABLE 1:** Tournament sample and outcome distribution by stage, complete tournament. Decided means a winner at the end of play; shootout matches are counted within draws.

Stage	Matches	Decided	Draws	Shootouts	Goals
Group Stage	72	52	20	0	215
Round of 32	16	13	3	3	42
Round of 16	8	7	1	1	23
Quarter-Finals	4	4	0	0	12
Semi-Finals	2	2	0	0	5
Bronze Final	1	1	0	0	10
Final	1	1	0	0	1
Total	104	80	24	4	308

### Comparisons between win and loss performances

#### Cliff’s delta effect sizes across the performance metrics

Eighty-six of 125 metrics differed between win and loss performances under the paired Wilcoxon test following false discovery rate control; the unpaired sensitivity analysis gave 83 significant metrics, 81 of them shared, with an identical leading group. The largest effect belonged to on-target attempts inside the penalty area (win 4.4, loss 1.7; delta = 0.68, q < 0.001), followed by total attempts on target (6.0 versus 2.7; delta = 0.66) and on-target attempts conceded (delta = -0.66). Possession (51.0% versus 40.4%; delta = 0.59) came next; the two values do not sum to 100% because FIFA assigns the remaining share, 8.6% on average, to contested possession. Distributions completed under pressure (230 versus 173; delta = 0.56), passes completed (482 versus 358; delta = 0.56) and ball receiving between the midfield and defensive lines (106 versus 81; delta = 0.52), receptions between the lines, joined the leading group. Ball recovery time also discriminated (14.7 s versus 18.6 s; delta = -0.53). Defensive pressures applied ran opposite to intuition: losing teams pressed more (295 versus 231; delta = -0.56). Figure 1 presents the ranked effect sizes.

**FIG. 1:**
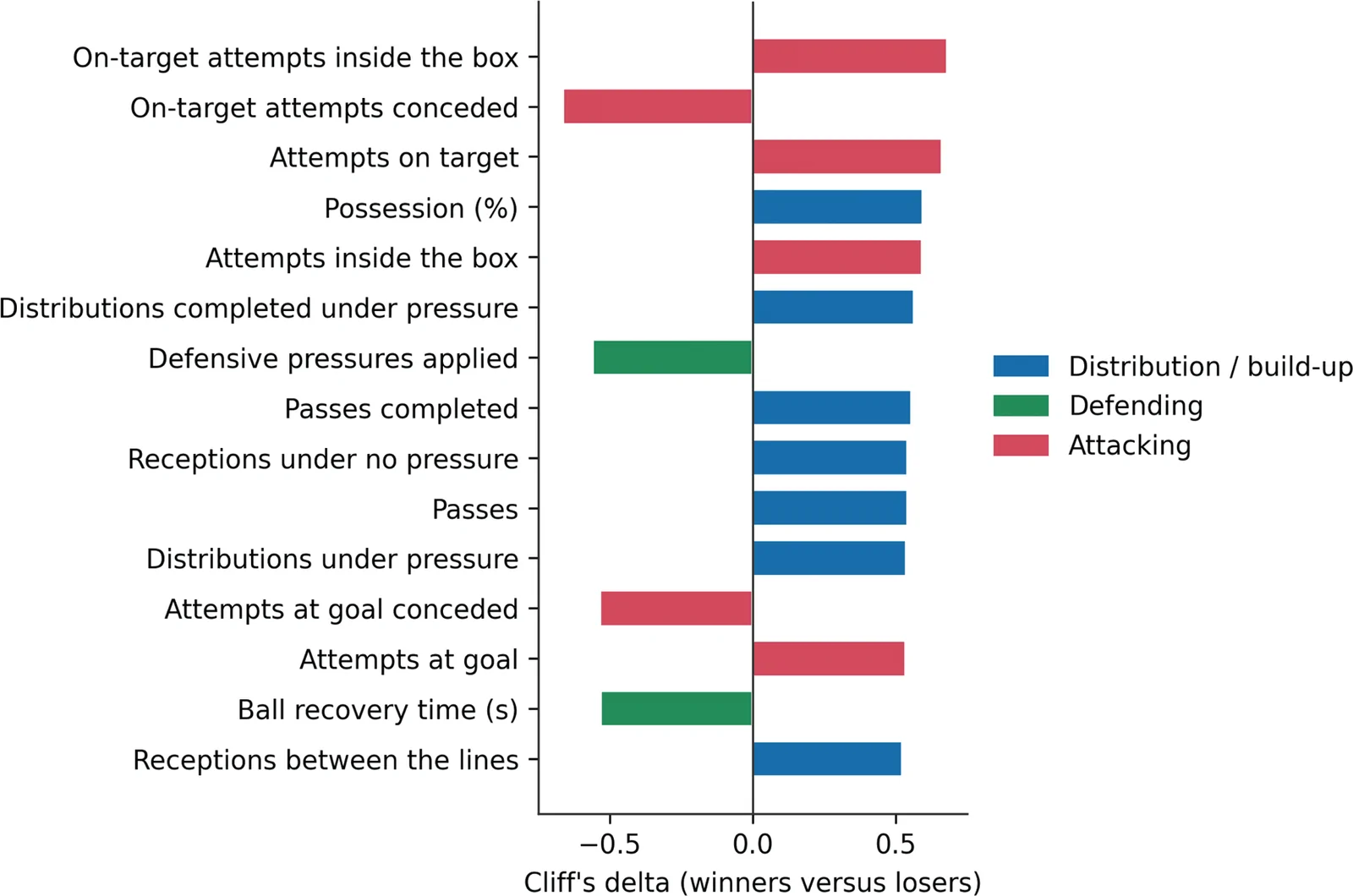
Cliff’s delta effect sizes for the fifteen leading performance metrics, winning versus losing team-match performances, all significant after false discovery rate control (q < 0.05). Colour encodes metric family. Cliff’s delta ranges from -1 to 1, with positive values indicating higher values in winners; q, false discovery rate adjusted probability.

#### Stage trajectories of the leading metrics

Figure 2 tracks the five leading metrics across the tournament for winners and losers, with the semi-finals, bronze final and final grouped as the finals stage. The winner-loser gap in on-target attempts inside the penalty area and in distributions completed under pressure persisted at every stage, with the distributions gap widest in the quarterfinals.

**FIG. 2:**
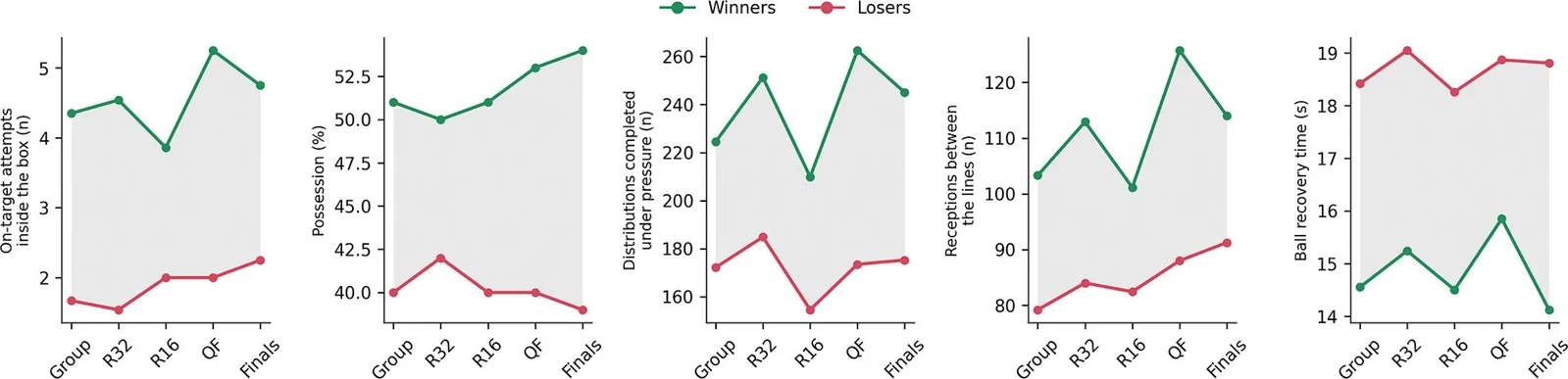
Stage trajectories of the five leading metrics for winners (green) and losers (red); the shaded ribbon is the winner-loser gap. Semi-finals, bronze final and final are grouped as the finals stage. Units are shown on each panel vertical axis.

### Classification performance across algorithms and specifications

In the primary differential specification (80 matches, 99 features), logistic regression reached an area under the curve of 0.946 ± 0.053 (median 0.953, interquartile range 0.922 to 0.984 across 25 held-out folds) with accuracy 0.875. The support vector machine reached 0.910 ± 0.077, the neural network 0.896 ± 0.080, random forest 0.892 ± 0.086 and gradient boosting 0.821 ± 0.100, rising to 0.881 ± 0.086 with nested hyperparameter tuning. Because features exceed matches in this specification, discrimination is read descriptively, with the effect-size analysis primary. As a post hoc descriptive check, a differential logistic model restricted to the five key duel metrics defined under Winning profiles: factor counts, thresholds and profile clustering reached 0.912 ± 0.078. The five metrics were selected from the leading effect sizes of the complete dataset, outside the cross-validation loop. This estimate may therefore be optimistic and is not independent evidence that discrimination holds without the high-dimensional feature set. In the paired specification (160 team-match rows), logistic regression reached 0.919 ± 0.032 (accuracy 0.831, F1 0.833), the neural network 0.899 ± 0.036, the support vector machine 0.892 ± 0.044, gradient boosting 0.859 ± 0.051 and random forest 0.853 ± 0.055. The shallow neural network matched but never exceeded the regularised linear model. Three-class accuracy reached 0.617 against a 0.388 majority baseline, with draws the dominant error source. A sensitivity analysis excluding the five matches carrying the source-feed attempt anomaly left discrimination and the leading effects unchanged (differential logistic area under the curve 0.946 ± 0.053 in the full set versus 0.945 ± 0.047 without them; on-target attempts inside the box delta = 0.68 in both), and the averaged SHAP leader was unchanged. A further sensitivity analysis excluding the five matches decided in extra time also left the leading effects and benchmarks materially unchanged (on-target attempts inside the box delta = 0.678 versus 0.681; 50% and 75% thresholds 2.8 and 4.3 versus 2.9 and 4.3; area under the curve 0.966 ± 0.043 on the 75 remaining matches). Figure 3 compares the cross-validated distributions.

**FIG. 3:**
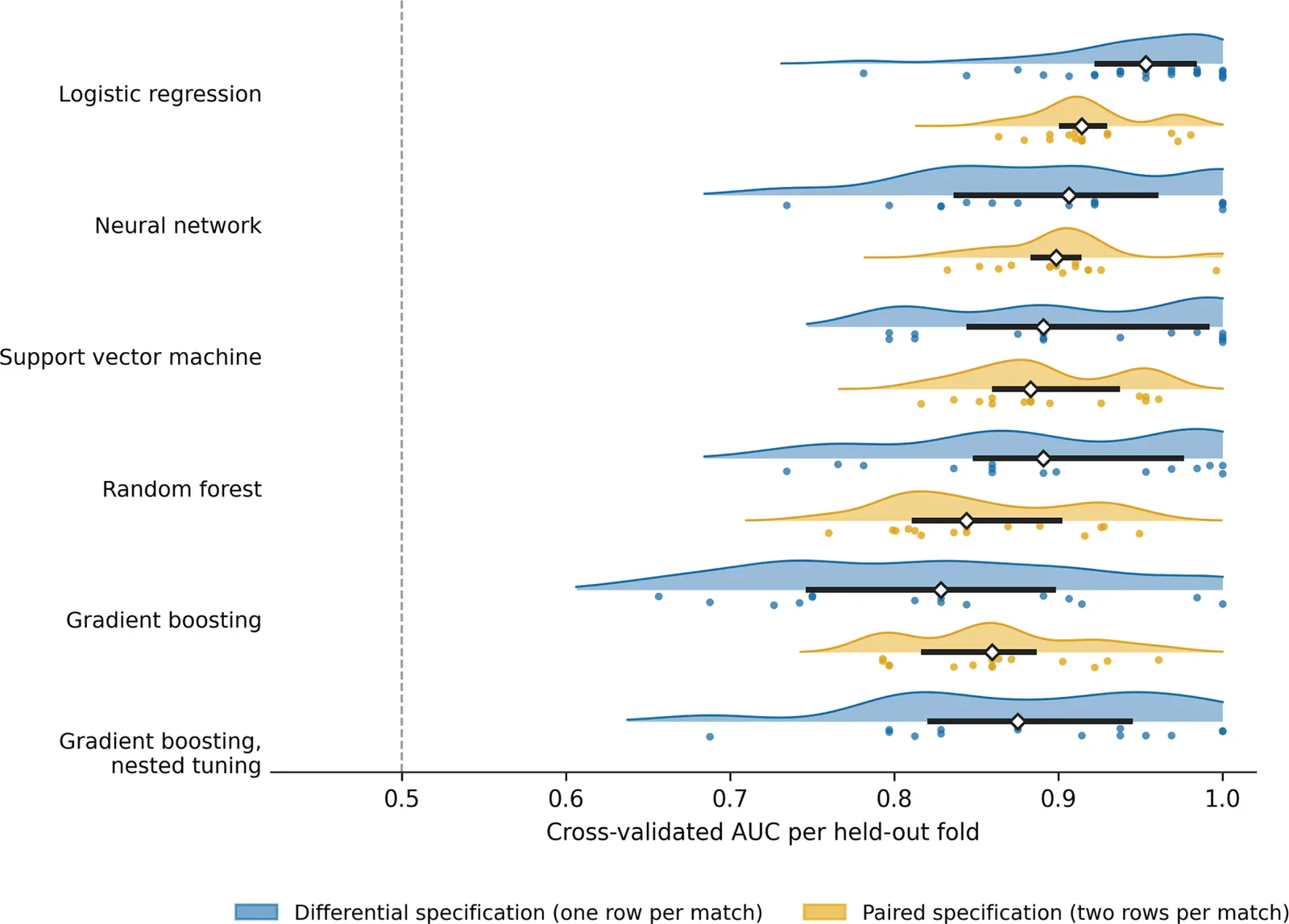
Raincloud comparison of cross-validated discrimination across algorithms and specifications. For each model, the half-violin shows the density of held-out fold AUC values, the points show the individual folds, the black bar spans the interquartile range and the diamond marks the median. Blue, differential specification (one row per match); amber, paired specification (two rows per match). Dashed line, chance discrimination. AUC, area under the receiver operating characteristic curve.

### SHAP analysis of the differential gradient boosting model

#### Global SHAP importance

Within the gradient boosting model, walking distance carried the largest mean absolute SHAP value (3.92), followed by on-target attempts inside the penalty area (1.81), total attempts on target (1.31), receptions between the lines (0.40), distributions completed under pressure (0.33) and defensive pressures applied (0.33). The importance mass concentrated heavily on the leading features: individual reference assignments agreed with the averaged ranking only moderately (median Spearman rho = 0.52, range 0.27 to 0.64), so positions below the leading features should be read as descriptive rather than inferential. The averaged leader is one plausible top feature rather than a settled ranking. Figure 4 shows the SHAP summary.

**FIG. 4:**
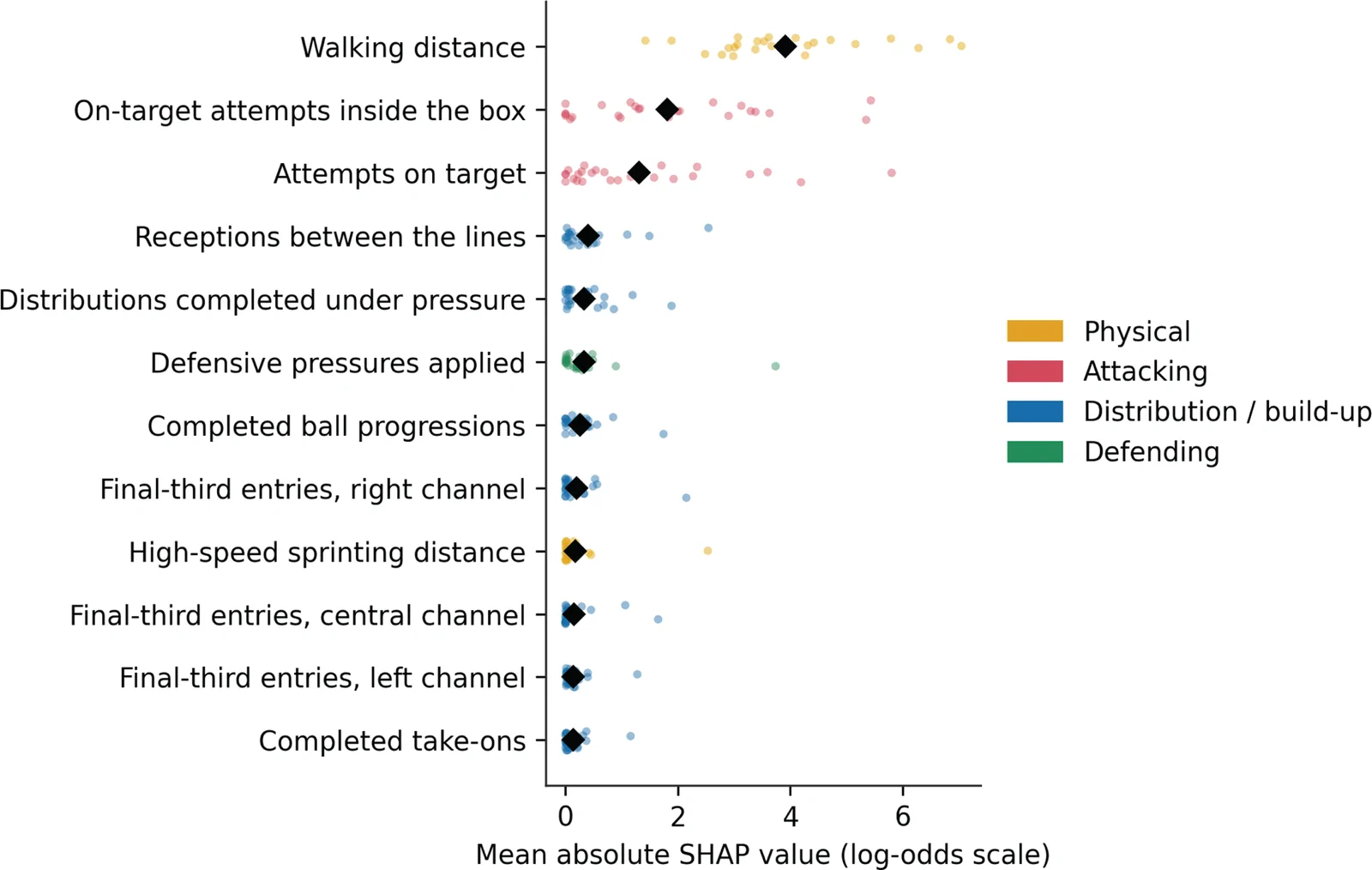
Mean absolute SHAP values of the twelve leading features in the differential gradient boosting model. Diamonds, average across 25 random reference-side assignments; circles, individual assignments; colour encodes metric family. SHAP, SHapley Additive exPlanations.

#### SHAP interaction structure

The strongest pairwise interaction joined walking distance with total attempts on target (mean absolute interaction 0.79), closely followed by walking distance with on-target attempts inside the penalty area (0.71). Walking distance also interacted with receptions between the lines (0.48) and with distributions completed under pressure (0.25), so every leading interaction ran through the walking-distance channel. Figure 5 maps the interaction matrix.

**FIG. 5:**
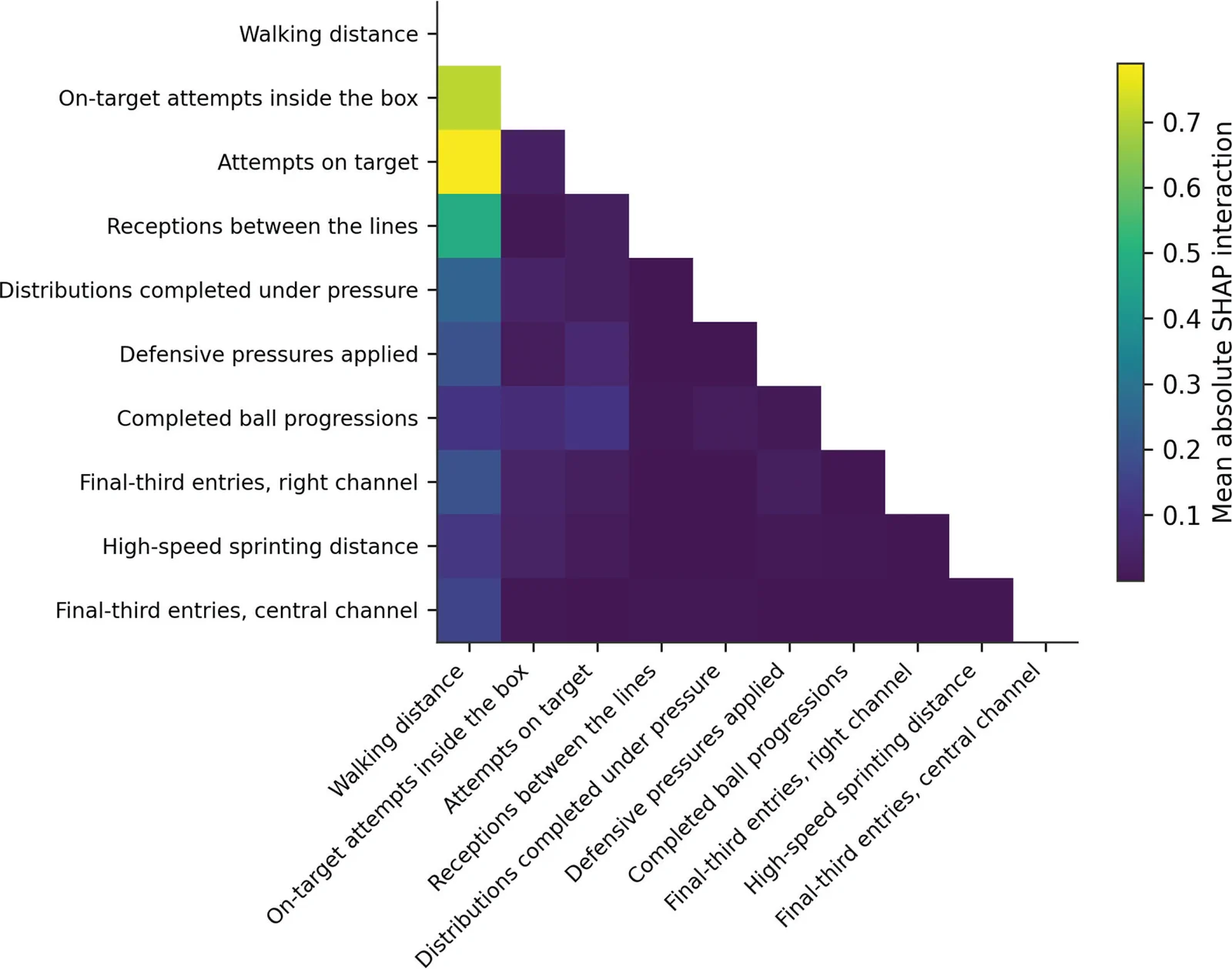
Mean absolute SHAP interaction values among the ten leading metrics of the differential model, averaged across 25 random reference-side assignments. The matrix is symmetric, so the lower triangle is shown. SHAP, SHapley Additive exPlanations.

#### SHAP dependence of the leading pair

Figure 6 resolves the leading interaction at match level. The SHAP contribution of the walking-distance differential rose steeply once a team out-walked its opponent, and the contribution was amplified when the same team also created more attempts on target. The mirror panel shows the attempts-on-target differential contributing most when paired with superior walking distance, the statistical signature of controlled tempo converted into chance quality.

**FIG. 6:**
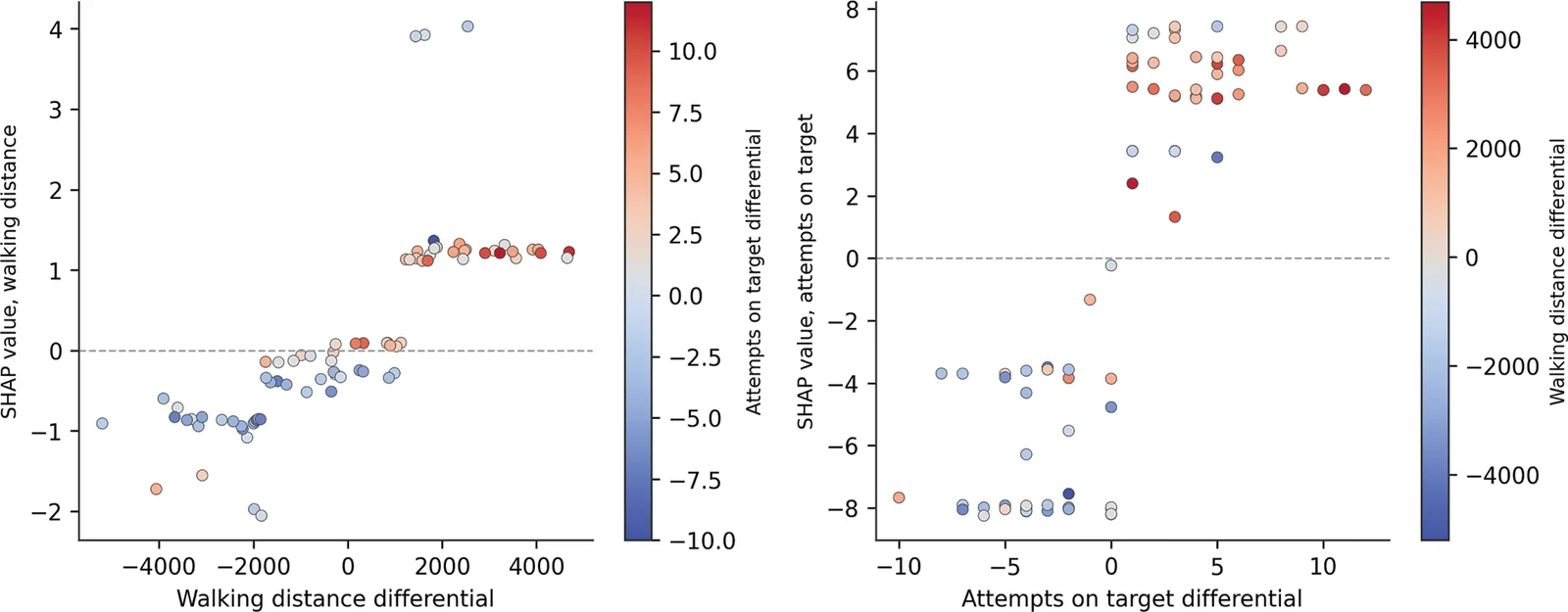
SHAP dependence of the leading interaction pair in the differential model, shown for one representative reference assignment. Left, walking distance coloured by attempts on target. Right, attempts on target coloured by walking distance. SHAP, SHapley Additive exPlanations; differentials are team value minus opponent value, in metres for walking distance.

### Logistic threshold estimates for the leading metrics

Win probability crossed 50% at 2.9 on-target attempts inside the penalty area and 75% at 4.3 (area under the curve 0.840). The corresponding values were 4.1 and 6.1 for total attempts on target (0.832), and 200 and 258 for distributions completed under pressure (0.782). Receptions between the lines crossed at 93 and 120 (0.761), completed passes at 419 and 548 (0.778), and completed line breaks at 109 and 144 (0.734). Ball recovery time ran in reverse: 16.5 s for 50% and 12.2 s for 75% (0.767). These benchmarks are calibrated on decided matches and read as conditional on a match producing a winner. Table 2 lists the benchmark values and Figure 7 presents the probability curves.

**TABLE 2:** Practical benchmarks from univariate logistic curves: metric values at which win probability crosses 50% and 75%, with univariate discrimination. AUC, area under the receiver operating characteristic curve; P(win), probability of winning. Ball recovery time improves as it decreases.

Metric	Value at P(win) = 50%	Value at P(win) = 75%	AUC
On-target attempts inside the box	2.9	4.3	0.840
On-target attempts, total	4.1	6.1	0.832
Distributions completed under pressure	200	258	0.782
Receptions between the lines	93	120	0.761
Passes completed	419	548	0.778
Completed line breaks	109	144	0.734
Ball recovery time (s)	16.5	12.2	0.767

**FIG. 7:**
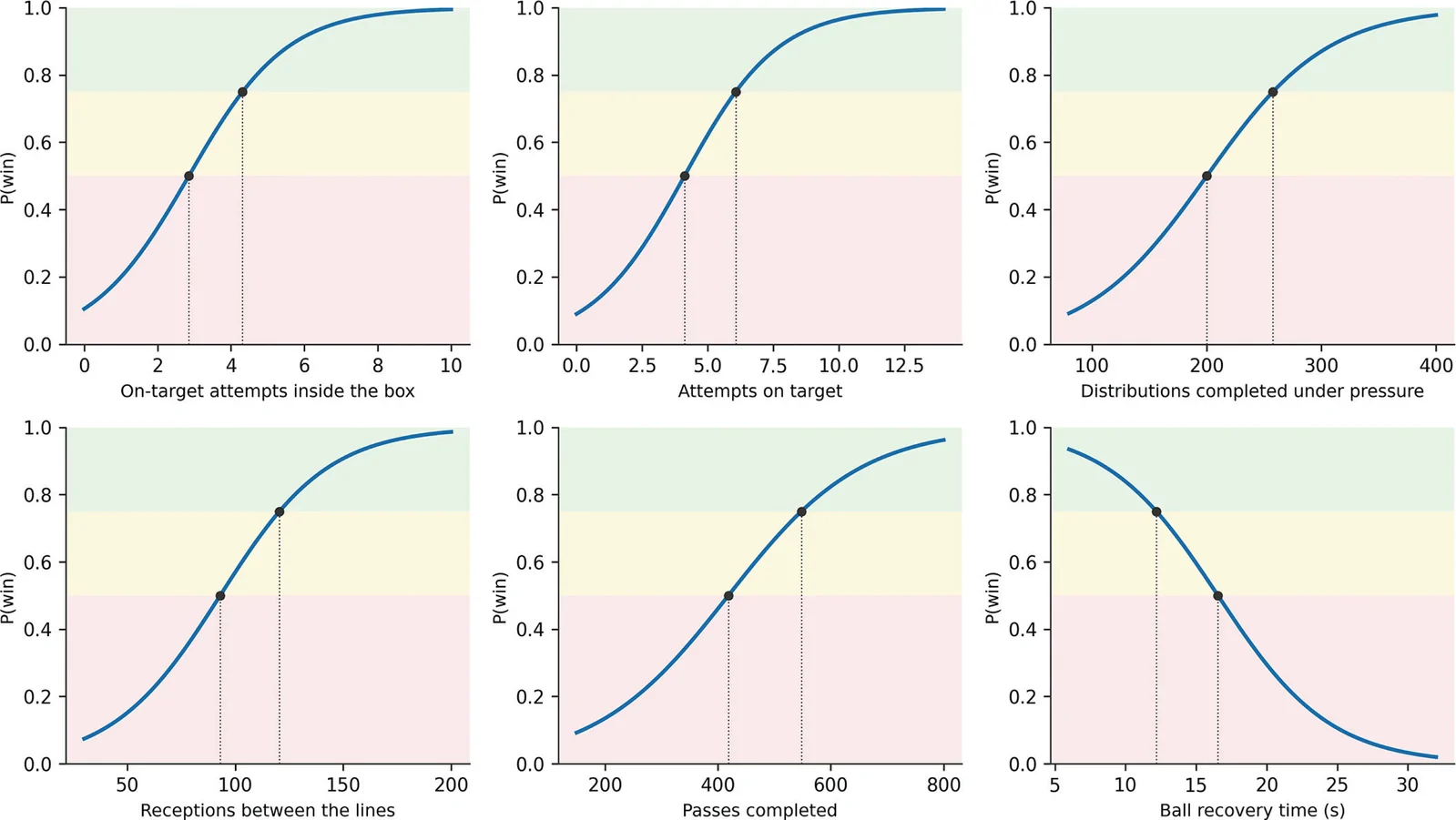
Univariate logistic win probability curves for six leading metrics with 50% and 75% crossings marked. Background shading: below 50%, 50 to 75%, above 75% win probability. P(win), probability of winning the match.

### Multi-metric combinations

#### Win probability by number of dominated key metrics

Teams that won all five key duels (on-target attempts inside the box, possession, distributions completed under pressure, receptions between the lines, faster ball recovery) won 45 of 49 such performances (92%). Winning four of five gave 77% (10 of 13). Winning none gave 13% (7 of 53). The zero-duel and five-duel groups are not mirror images (53 versus 49 performances) because a tied metric value awards the duel to neither team; eight decided matches carried at least one tied duel metric, seven of them on on-target attempts inside the box. Figure 8 shows the dose-response pattern, although the intermediate groups are small and their intervals overlap.

**FIG. 8:**
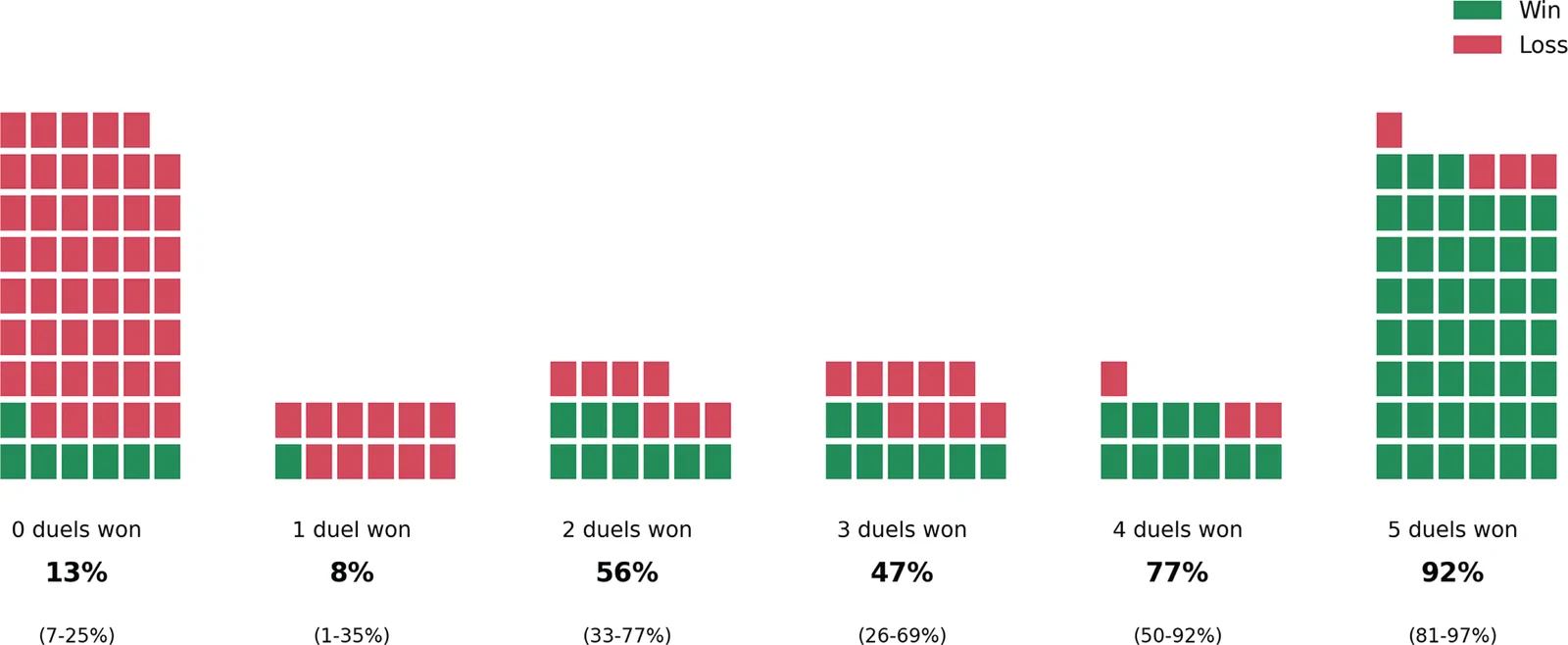
Every decided team-match performance (n = 160) as one tile, grouped by the number of the five key duels won against the opponent, from zero to all five (on-target attempts inside the box, possession, distributions completed under pressure, receptions between the lines, faster ball recovery; defined under Winning profiles: factor counts, thresholds and profile clustering). Win percentages below each group. CI, 95% confidence interval (Wilson method).

#### Paired-metric synergy and decision rules

Ball recovery time appeared in every leading synergy pair. Its combination with receptions between the lines gave the largest gain over the best single member (+0.037, pair area under the curve 0.807), and its combination with on-target attempts inside the box produced the highest paired discrimination (0.865). A depth-two decision tree reproduced 74.8% ± 5.5% cross-validated accuracy with two rules: with possession above 45%, more than 3.5 attempts on target classified a win; with possession below 45%, holding the opponent under 3.5 on-target attempts classified a win. Figure 9 presents the synergy matrix and the tree.

**FIG. 9:**
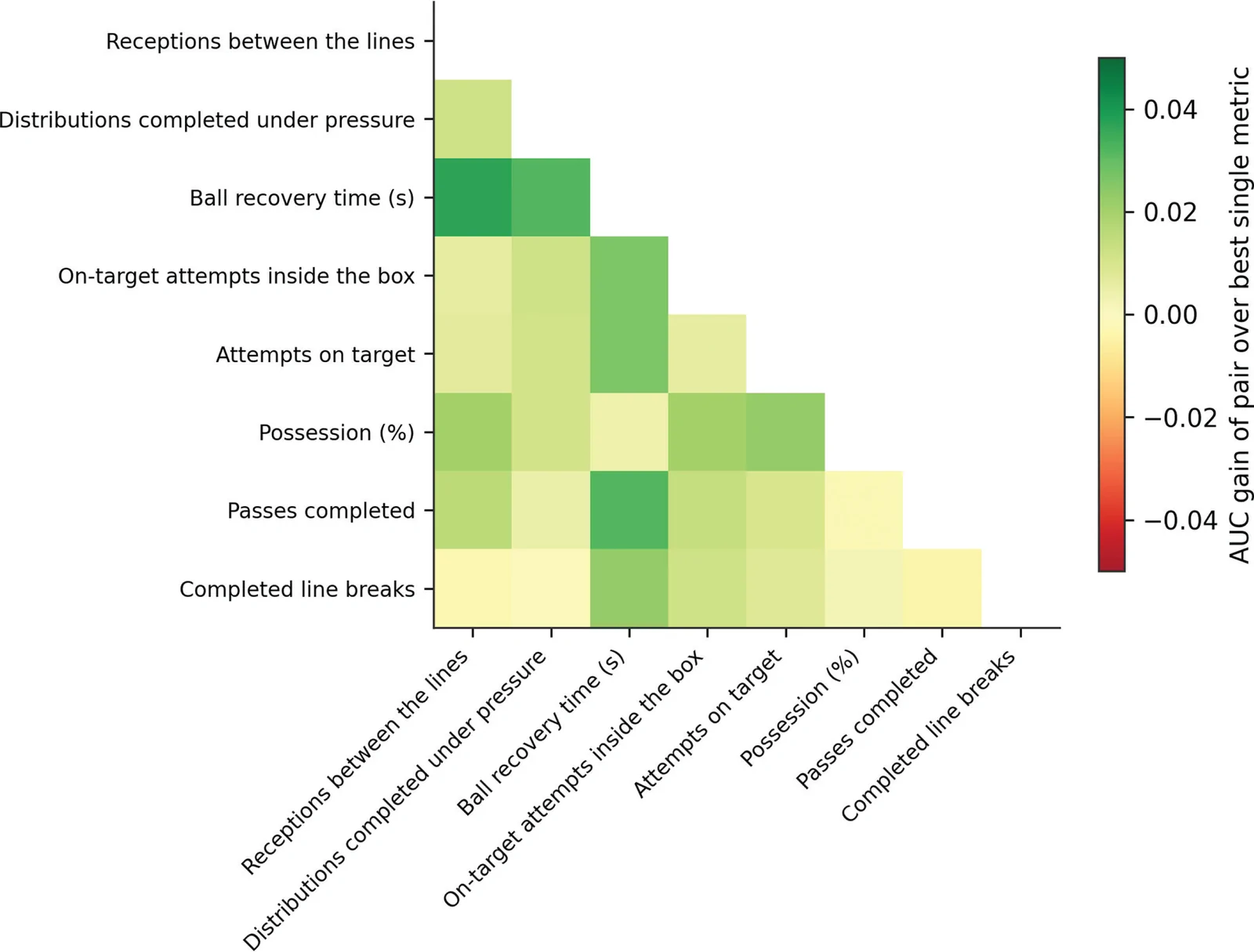
Paired-metric synergy: cross-validated AUC gain of each two-metric model over its best single member. The matrix is symmetric, so the lower triangle is shown. AUC, area under the receiver operating characteristic curve.

### K-means clustering of winning performances

Two clusters maximised silhouette, although the value (0.20 at k = 2) indicates weak and largely overlapping separation, so the two groups are read as descriptive winning profiles rather than distinct types. Repeating the clustering on 50 random ten-metric subsets of the fifteen style metrics reproduced the same structure (median adjusted Rand index 0.64), and the proactive pattern of higher possession with faster ball recovery appeared in every subset. The proactive profile (38 wins) combined high possession, completed line breaks and receptions between the lines with fast recovery and minimal low-block time. The reactive profile (42 wins) combined a low defensive block, slower ball recovery and more counterattacking play with below-average possession and line breaks. Spain (six wins, the final included) and Argentina (five) concentrated in the proactive cluster, while England, France and Mexico contributed the most reactive wins. Both finalists, including the champion Spain, belonged to the proactive cluster. Figure 10 shows the standardised profile patterns.

**FIG. 10:**
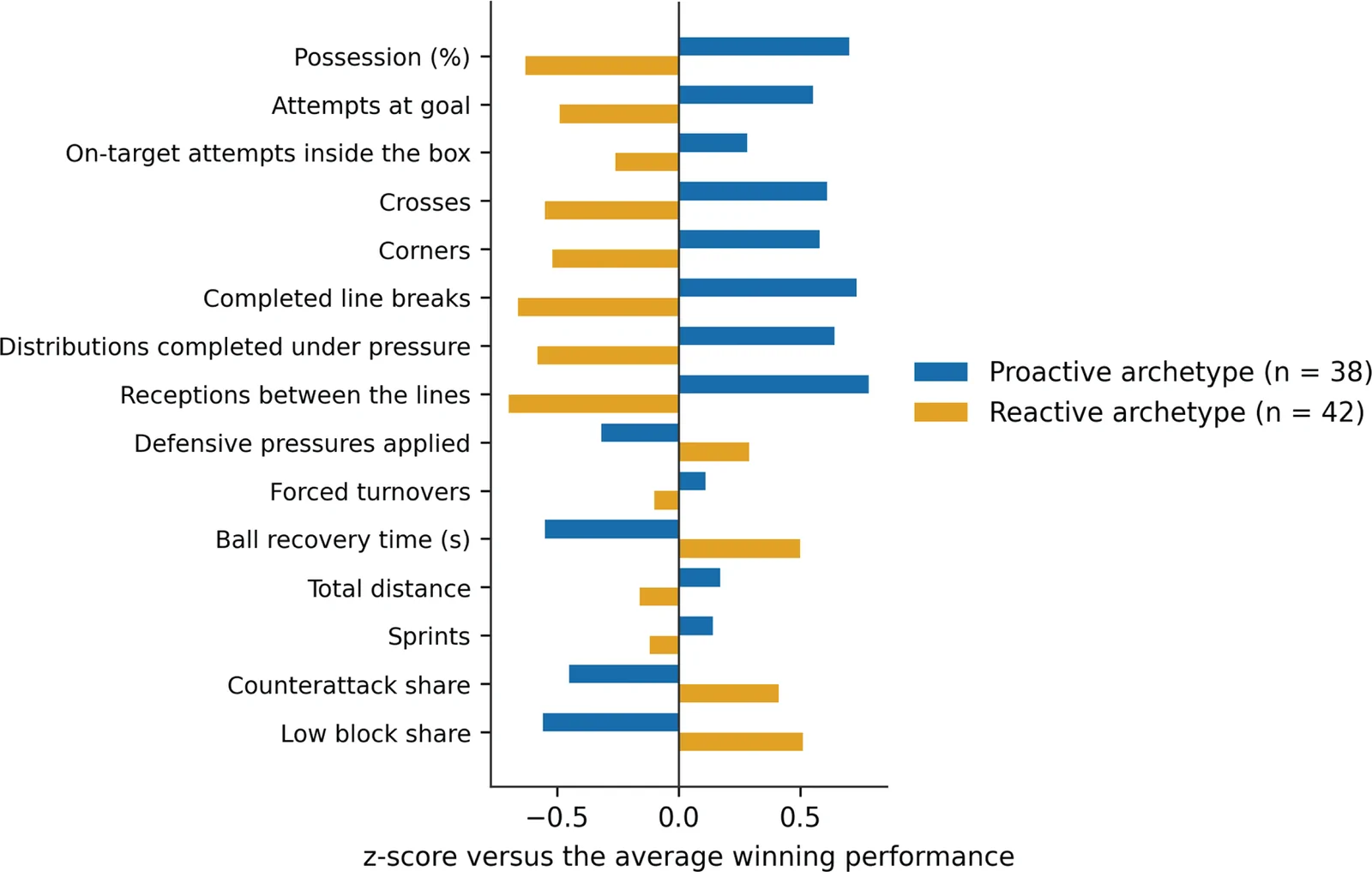
Standardised metric profiles of the two descriptive winning profiles. z-scores express each metric in standard deviations from the mean of all winning performances.

### Stratified models

#### Group stage versus knockout rounds

Logistic discrimination held in both strata (group: 0.914 ± 0.068, n = 104 rows; knockout: 0.887 ± 0.101, n = 56 rows). The determinant hierarchy shifted. Possession sat among the five leading group-stage effects (delta = 0.58) but dropped out of that leading set in the knockout rounds, where distributions completed under pressure entered it (delta = 0.65) and on-target attempts inside the box strengthened from delta = 0.65 to 0.75. Figure 11 presents both strata.

**FIG. 11:**
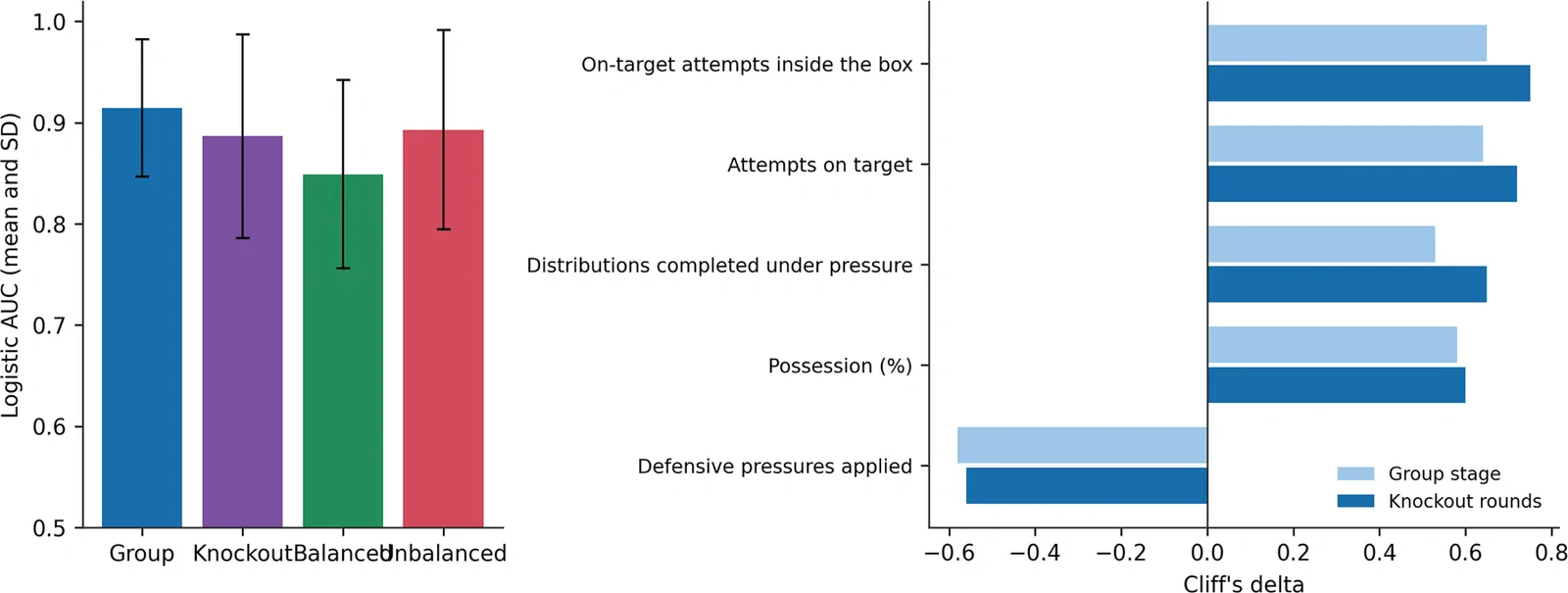
Stratified analysis. Left, logistic discrimination per stratum. Right, effect-size shift of the leading metrics from group stage (light) to knockout (dark). AUC, area under the receiver operating characteristic curve; SD, standard deviation.

#### Balanced versus unbalanced pairings

In unbalanced matches (pot gap 2 to 3; 37 decided), winners dominated across the board (model area under the curve 0.893 ± 0.098). Deltas reached 0.84 for on-target attempts inside the box, 0.81 for completed defensive line breaks, 0.81 for distributions under pressure and 0.81 for possession. In balanced matches (pot gap 0 or 1; 43 decided), the discriminating set narrowed to finishing precision: 0.53 for on-target attempts inside the box and -0.54 for on-target attempts conceded, with walking distance (0.42) and defensive pressures applied (-0.41) the only other effects above 0.40 in absolute value (area under the curve 0.849 ± 0.093).

### Conformal prediction analysis

Across 20 repeated held-out evaluations per level, empirical coverage tracked the nominal confidence level across the whole range tested (75% to 95%), sitting slightly below it at every level, averaged 0.885 at the 90% target, and was therefore close to the nominal target within the observed sampling variability. Efficiency followed the expected trade-off: 89% of test matches received a confident single-outcome prediction at the 85% level, 85% at the 90% level, and 61% at the 95% level, with 15% flagged at the 90% level as statistically uncertain from performance data alone. Figure 12 shows the validity and efficiency curves.

**FIG. 12:**
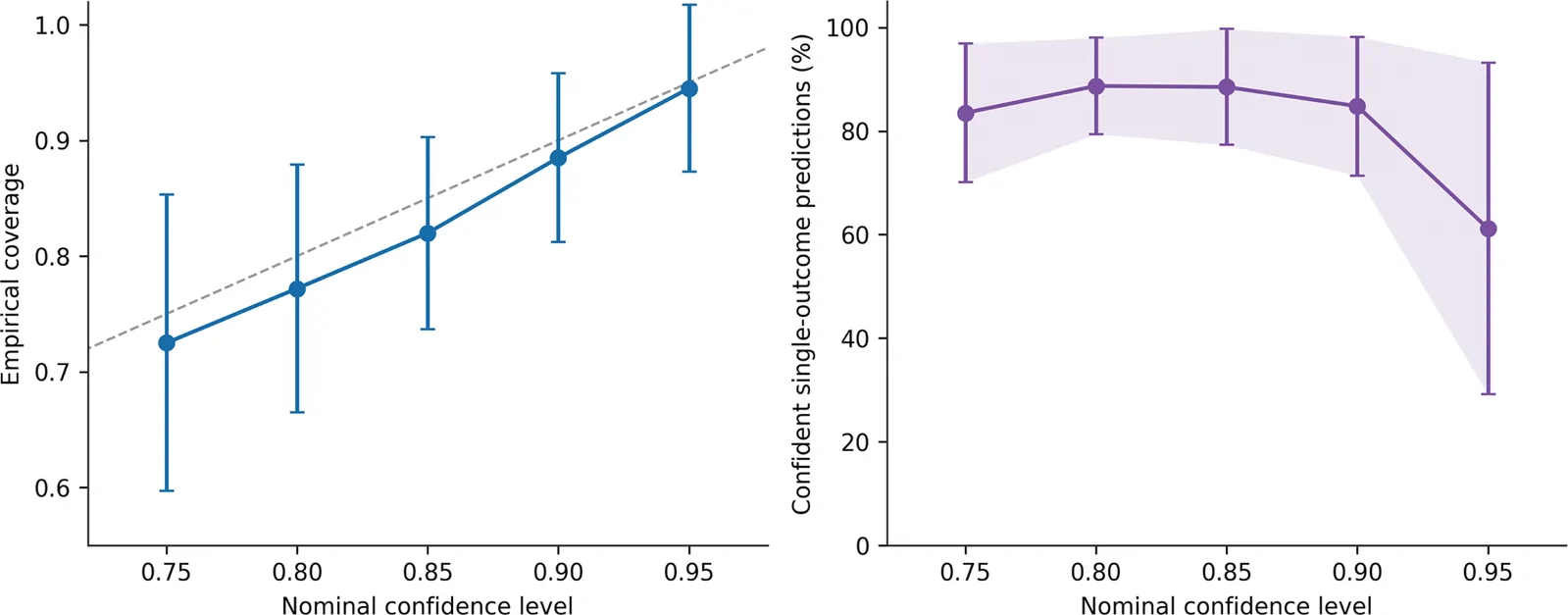
Conformal validity and efficiency across nominal confidence levels (75% to 95%), 20 held-out evaluations per level. Left, empirical coverage against the diagonal identity line; coverage tracks the nominal level within sampling variability. Right, share of confident single-outcome predictions; informativeness declines as the confidence demand rises. Error bars and bands, standard deviation across repetitions.

## DISCUSSION

### Main findings

We analysed the first 48-team World Cup with the complete official FIFA metric set and a modern explainable pipeline. Four findings stand out. On-target attempts inside the penalty area were the strongest single discriminator of winning. Tempo control, expressed as walking distance, carried the largest model importance, and composure under pressure, led by distributions completed under pressure, grew in weight as stakes rose. Winning performances split into two descriptive profiles, proactive and reactive, yet both profiles depended on the same two capacities. And the expanded format left the core determinants intact while reshaping their hierarchy across stages and pairing balance.

### On-target attempts inside the penalty area as the primary discriminator

The delta of 0.68 for on-target attempts inside the box extends a consistent line of evidence. Castellano et al. [4] identified shots on target as the metric that best discriminated successful teams across three World Cups, and Liu et al. [5] confirmed its dominant effect on outcome probability at the 2014 edition. At the 2022 tournament, the SHAP analysis of Song et al. [10] ranked attempts on target and shooting opportunities as the two most influential features. Our contribution is the location term: the threshold analysis showed that roughly three on-target attempts inside the box gave even odds and four gave 75% among matches producing a winner, while shots from outside carried little weight, consistent with shot-location value analyses of expected goals [33]. The practical reading is direct. Chance quality inside the box, not shot volume (including distance shots), decided this tournament.

### Composure under pressure and the control of tempo

The enhanced feed changed the picture at the top of the model. Walking distance led the averaged SHAP ranking of the gradient boosting model, ahead of on-target attempts inside the box, and its interactions with the two finishing metrics were the strongest pairs in the tournament. This refines the 2022 hierarchy, where attempts on target and shooting opportunities led the SHAP ranking [10]. Finishing precision kept its central place, while the strongest model signal became the physical signature of match control. The new pressure metrics behaved as hypothesised in the direct comparisons: winners completed more distributions under pressure (delta = 0.56) and more receptions between the lines (delta = 0.52), and the distributions effect strengthened to delta = 0.65 in the knockout rounds. Winners walked more and passed better while pressed. We read this as tempo control: dominant teams slowed the game, conserved energy and kept technical security when opponents engaged them, a pattern that classic physical analyses could not see because distance alone was reported to poorly discriminate outcome in isolation [8, 34]. The same logic explains why receptions between the lines and completed line breaks joined the leading effects. These metrics quantify the willingness to receive in dangerous space under pressure, a quality earlier tournaments could only infer indirectly [13]. Sprint-type outputs mattered mainly through goal situations, where straight sprinting remains the most frequent action [35], but they did not clearly separate winners from losers overall. Walking distance may also partly index a leading scoreline, because teams protecting a lead manage tempo, so the tempo-control reading carries the same reverse-causation caution as every observational metric here.

### The pressing paradox

Losing teams applied more defensive pressure (295 versus 231 events; delta = -0.56) and recovered the ball more slowly (18.6 s versus 14.7 s). This inverts a naive reading of pressing effectiveness and matches the game-state literature: teams that fall behind chase the ball, so high pressing volume often marks the loser rather than a winning pattern [36, 37]. Effective pressing appears in our data as fast ball recovery, not as pressure volume, in line with ball recovery studies in elite leagues [38, 39]. Data-driven counter-pressing detection points the same way, valuing the speed and location of the regain over the number of pressing events [40]. The inversion appeared in every stratum and was strongest in unbalanced pairings (delta = -0.72, against -0.41 in balanced pairings), so falling behind against a stronger opponent exaggerates the chasing pattern. Analysts should therefore read raw pressing counts with the score state in mind.

### Combinations, thresholds and the two routes to victory

The combination analyses answered a question single-metric rankings cannot. Dominating all five leading duels, on-target attempts inside the box, possession, distributions completed under pressure, receptions between the lines and faster ball recovery, gave a 92% win rate, and dominating none gave 13%, a dose-response profile that turns abstract importance values into a scouting checklist. Ball recovery time acted as the universal amplifier, improving every metric it was paired with, most strongly finishing precision (pair area under the curve 0.865 with on-target attempts inside the box). The depth-two tree condensed the tournament into two doors: hold possession above 45% and create at least four on-target attempts, or concede the ball and hold the opponent under four. Set-play attention remains warranted in knockout football [41], but neither door opened without one of the two core capacities. This two-door structure matches the two-profile result and echoes success-factor modelling at earlier tournaments, where efficiency variables outranked style variables [6, 9].

### Did the expanded tournament format change the rules?

Partly, and in an informative direction. The core determinants survived the format change, so the 32-team evidence base remains relevant. Their hierarchy did not survive. Possession led the group-stage hierarchy but lost its leading place in the knockout rounds, where pressure-resistance metrics entered the leading set and finishing precision strengthened further. In unbalanced pairings, everything separated winners from losers, the statistical signature of dominance in mismatches that the seeding literature predicted for a 48-team field [14, 15]. In balanced pairings, only finishing precision retained a large effect. The 17% upset rate, computed over the 71 decided cross-pot pairings reported under Match sample and descriptive statistics and therefore on a different denominator from the balanced and unbalanced strata, quantifies the competitive risk carried by the expanded draw [16]. For coaches of lower-ranked teams the message is encouraging: against stronger opponents the controllable margin lives almost entirely in box efficiency at both ends, and heat management around a three-country summer tournament adds a further controllable layer [42]. Preparation lessons from recent major tournaments, from pandemic-era planning for the 2022 FIFA World Cup to player-welfare considerations in rapidly professionalising leagues, widen this planning context for federations [43, 44, 45].

### Methodological position relative to the 32-team era

Three design choices distinguish this analysis from the 2022 studies [10, 11]. The differential specification removed the paired-row dependence that inflates apparent sample size in two-row designs. Conformal analysis attached calibrated uncertainty to every prediction, an honesty layer machine learning applications in sport have generally lacked [20, 46], and 15% of held-out matches proved genuinely uncertain from performance data alone, consistent with the irreducible aleatoric component of match outcomes [46]. And explanation moved from importance rankings to interaction structure, following the direction that football machine learning reviews have called for [3, 47]. A shallow neural network was added as a representation-learning benchmark and matched, but never exceeded, the regularised linear model. With 80 decided matches, the data density is too low for representation learning to find structure beyond what a regularised linear combination of the same features captures, so the added flexibility of the network buys variance rather than signal. This supports interpretable learners as the honest choice at tournament sample sizes.

### Practical applications

Four applications follow from the observed associations; all describe discrimination in an observational census and are proposed as testable training priorities, not established causal levers. Training design could weight finishing inside the box under match-realistic pressure, because four on-target attempts inside the box marked the 75% win threshold among decided matches. Possession work could prioritise pressure resistance over sterile circulation, targeting the roughly 260 completed distributions under pressure that marked the same conditional threshold. Transition coaching could emphasise recovery speed, aiming under a 12 s ceiling, rather than pressing volume. And opposition analysis could profile opponents on the five-duel checklist, because winning four of five duels was followed by victory in more than three quarters of decided cases whatever the profile adopted. Looking ahead, autonomous artificial intelligence agents are beginning to restructure data collection and analysis pipelines in sports science [48], while critical evaluations of AI applications in applied sport settings show that their outputs still require expert oversight before they guide practice [49]. The five-duel checklist and the threshold benchmarks reported here are directly computable from the published FIFA feed, and their automation within such supervised systems is a natural next step for federation and league analytics departments.

### Limitations

Nine limitations deserve statement. First, team-level data cannot resolve individual contributions, so all findings describe collective behaviour. Second, the observational design identifies discrimination, not causation; winning also changes behaviour through game state. Third, draws resisted classification in the three-class model, a known ceiling for outcome modelling from performance data. Fourth, 80 decided matches limit statistical power in the strata, and knockout estimates carry wide intervals. Fifth, FIFA did not publish the advanced metric panel for one group match (Belgium versus Egypt, still unpublished as of 5 August 2026), which contributes its core panel to the descriptive analyses and is excluded from the multivariable models.

In the same feed, five source rows (matches 9, 20, 73, 85 and 103) carried a one-unit attempt anomaly; sensitivity analyses excluding these rows are reported. Sixth, the FIFA metric definitions, including woodwork handling in attempt categories, are proprietary and were taken as published. Seventh, the SHAP importance ranking concentrated on a small leading group and varied across random reference-side assignments; it was therefore averaged across 25 assignments and is treated as descriptive, with the effect-size analysis as the primary evidence. Eighth, match statistics aggregate the full match, extra time included, and no normalisation per 90 minutes was applied; five decided matches went to extra time, and excluding them left effects and thresholds materially unchanged, but per-minute rates could shift knockout comparisons at the margin. Finally, one tournament is a single sample of tournament football; replication at the 2030 edition will separate format effects from cohort effects.

## CONCLUSIONS

At the 2026 FIFA World Cup, the first ever tournament played by 48 teams, winning performances were most consistently associated with two characteristics. Precision inside the penalty area was the most consistent discriminator across every stratification we applied, and composure in possession under pressure showed strong overall and knockout-stage associations. Around four on-target attempts inside the box and around 260 completed distributions under pressure marked the 75% win probability lines among decided matches, and teams dominating the five leading duels won 92% of the time. Two descriptive winning profiles coexisted: proactive and reactive. Both finalists won proactively, and the champion, Spain, contributed the most proactive wins of the tournament, including the final. For practice, we suggest that, while preparing for major football tournaments, (i) coaches treat pressured finishing and pressure-resistant possession as leading training priorities, and (ii) analysts read ball recovery time ahead of raw pressing volume in match evaluation. These suggestions describe associations observed at one tournament and warrant prospective testing. The analysis code accompanies this article, every value is verifiable on the public FIFA match centre, and the verified full-tournament workbook is available from the first author, serving as the reference baseline for the 48-team era.

## Data Availability

The complete data package holds the panel, the workbook, the goal-event table, the source snapshot check-sums and the code. All underlying values are published by FIFA and can be verified match by match at the official match centre. Because the organised panel is derived from content accessed under the non-commercial licence in the FIFA terms of service, it is not redistributed publicly. The corresponding author shares the full package, with its checksums, on request for academic and non-commercial use, and the verification code travels with it under the same conditions. Dated evidence of those terms was archived on 20 July 2026. Source: https://www.fifa.com/en/match-centre (accessed 14 to 20 July 2026 and verified on 5 August 2026).
